# Patient backgrounds and outcomes of mechanically ventilated children treated in pediatric intensive-care units versus general intensive-care units in Japan: a retrospective cohort study using a national inpatient database

**DOI:** 10.3389/fped.2026.1881384

**Published:** 2026-07-29

**Authors:** Yoshio Sakurai, Nobuaki Michihata, Kohei Osada, Shingo Kobayashi, Wataru Sakamoto, Yuta Uchida, Kuniya Ishii, Hiroya Yokohari, Toshiaki Isogai, Hiroki Matsui, Kiyohide Fushimi, Kensuke Yoshimura, Hideo Yasunaga

**Affiliations:** 1 Department of Pediatric Critical Care Medicine, Saitama Medical Center, Saitama Medical University, Saitama, Japan; 2 Cancer Prevention Center, Chiba Cancer Center Research Institute, Chiba, Japan; 3 Department of Cardiology, Tokyo Metropolitan Tama Medical Center, Tokyo, Japan; 4 Department of Health Services Research, Graduate School of Medicine, The University of Tokyo, Tokyo, Japan; 5 Department of Health Policy and Informatics, Institute of Science Tokyo, Tokyo, Japan; 6 Department of Clinical Epidemiology and Health Economics, School of Public Health, The University of Tokyo, Tokyo, Japan; 7 Center for Next Generation of Community Health, Chiba University Hospital, Chiba, Japan

**Keywords:** general intensive care unit, in-hospital mortality, mechanical ventilation, outcome, pediatric intensive care unit

## Abstract

**Objective:**

Pediatric intensive care units (PICUs) are well developed in Western countries and have been linked to improved survival. However, evidence comparing the outcomes between PICUs and general intensive care units (GICUs) remains inconsistent. In Japan, limited PICU capacity raises concerns that many children receive critical care in GICUs. This study compared the clinical outcomes between these settings using a national inpatient database and propensity score overlap weighting analyses.

**Methods:**

We conducted a retrospective cohort study using a national Japanese inpatient database from July 2010 to March 2022. We included mechanically ventilated children aged ≤14 years admitted to ICUs and compared patient characteristics between those treated in PICUs and GICUs. In-hospital mortality was evaluated using overlap weighting analyses.

**Results:**

Of the 48,106 eligible patients, 36% received care in PICUs. After overlap weighting analyses, PICU care was associated with a significantly lower in-hospital mortality than GICU care (mean 2.73% vs. 3.42%; odds ratio, 0.79; 95% CI, 0.69–0.91; *p* = 0.001). PICU care was associated with longer overall hospital stays (mean 22.74 vs. 22.13 days; difference, +0.61 days; 95% CI, 0.30–0.93; *p* < 0.001) and longer ICU stays (mean 6.37 vs. 5.42 days; difference, +0.95 days; 95% CI, 0.82–1.09; *p* < 0.001).

**Conclusion:**

Most mechanically ventilated children in Japanese ICUs were treated in GICUs. Pediatric patient care in GICUs was associated with higher mortality relative to that in PICUs, underscoring the need for further centralization.

## Introduction

In Western countries, mechanical ventilation for critically ill patients, both adults and children, is typically provided in intensive care units (ICUs) to optimize human and material resources (1, 2). Pediatric intensive care units (PICUs) were first established in the 1950s and became widely disseminated in North America and Europe between the 1960s and 1970s. Currently, approximately one PICU bed is available per 20,000–40,000 children, a level of capacity that is associated with an improved pediatric survival (3, 4).

Although the effectiveness of PICUs is well recognized, direct comparisons of the outcomes between pediatric patients treated in PICUs and those treated in general ICUs (GICUs) remain limited. A five-year retrospective cohort study from Sweden (5) involving approximately 17,000 admissions reported differences in case mix between PICUs and GICUs, while the standardized mortality ratios based on the Pediatric Index of Mortality 2 (PIM2) were similarly low in both settings. In contrast, a two-year retrospective cohort study from Finland (6) including approximately 4,900 pediatric admissions demonstrated a significantly higher mortality among children treated in GICUs, with an odds ratio of approximately four. Furthermore, a 14-year retrospective cohort study from Australia and New Zealand (7) involving approximately 43,000 pediatric admissions found no overall difference in the adjusted mortality between PICUs and GICUs. However, during the study period, the survival rate of pediatric patients improved significantly over time in PICUs compared with GICUs.

In Asia, the development of PICUs has been delayed, and the available evidence remains limited. Countries such as China, India, Korea, and Pakistan did not begin establishing PICUs until the 1980s and 1990s (8–11). In Japan, the first PICU was established in 1994, approximately 30 years later than in Western countries. Currently, PICUs are concentrated primarily in metropolitan areas, and more than half of Japan's prefectures (26 of 47) have no PICU. Consequently, many mechanically ventilated children receive intensive care in general wards or GICUs (12).

In this context, the current status of mechanically ventilated pediatric patients treated in PICUs or GICUs remains unclear in real-world clinical settings in Japan. Therefore, this study aimed to compare the clinical outcomes of mechanically ventilated children treated in PICUs and GICUs using a national inpatient database. Propensity score overlap weighting was used to adjust for differences in patient characteristics.

## Subjects and methods

### The study design and data source

This retrospective cohort study used the Japanese Diagnosis Procedure Combination (DPC) inpatient database, which includes discharge abstracts and administrative claims data from over 1,700 acute care hospitals throughout Japan (13). These hospitals voluntarily contribute to the database, encompassing each inpatient's demographic information and diagnoses, including age, sex, International Classification of Disease 10th Revision (ICD-10) codes, daily procedures, daily medication administration, and admission and discharge status. In 2017, the database covered approximately 75% of all ICU beds in Japan (14). A previous validation study demonstrated that the DPC database has high specificity and moderate sensitivity for diagnoses, as well as high specificity and sensitivity for the procedures (15).

### Study population

**Figure 1:**
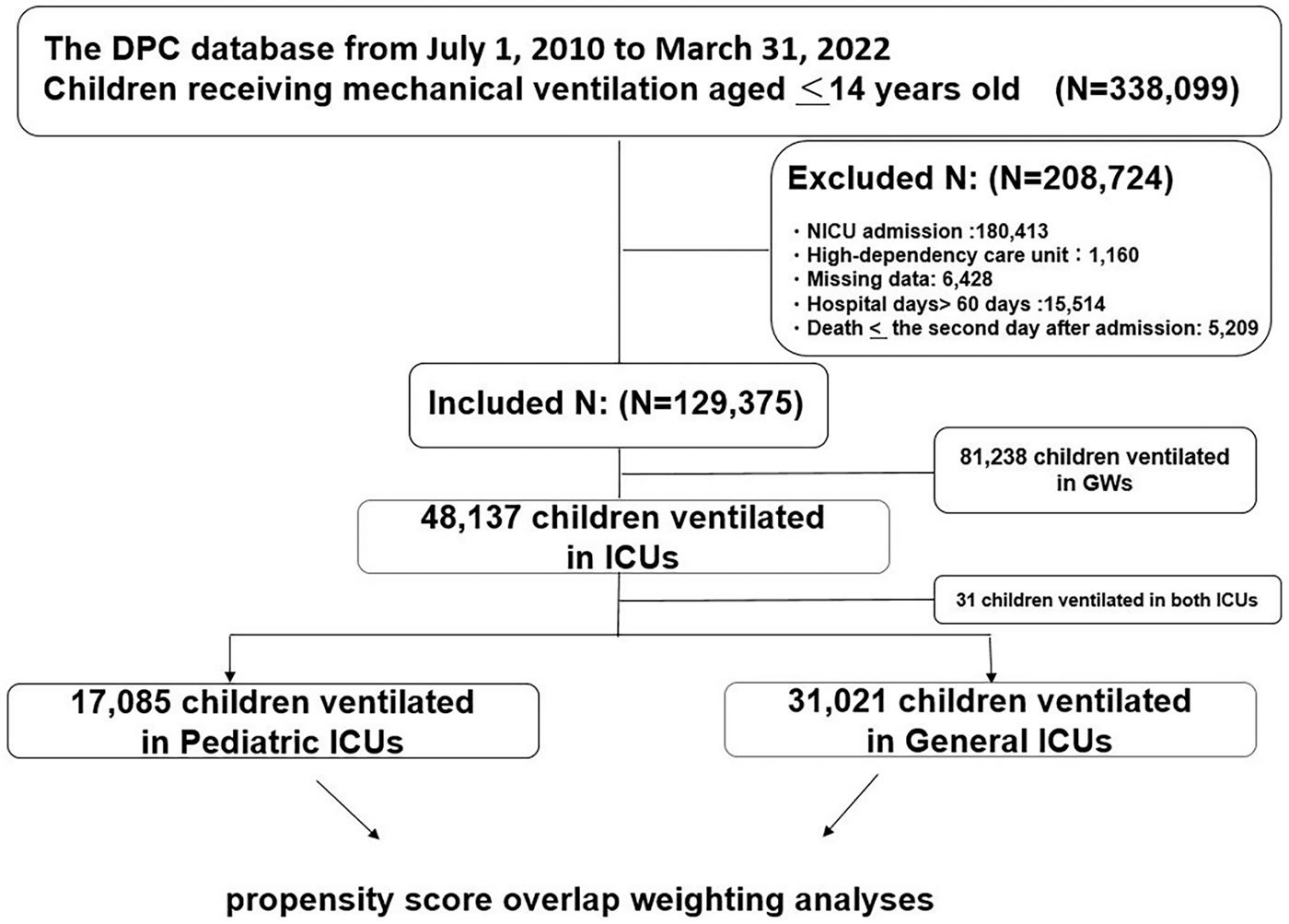
Flowchart of mechanically ventilated patient selection. GW, general ward; ICU, intensive-care unit.

We identified mechanically ventilated children aged ≤ 14 years using the DPC database from July 1, 2010, to March 31, 2022. Patients were excluded based on the following criteria: (1) admission to neonatal intensive care units, (2) admission to high-dependency care units, (3) missing information regarding the ward to which patients were admitted, (4) hospital stay longer than 60 days, and (5) death within the first 2 days of admission (Figure 1).

High-dependency care units are defined as hospital units where the level of patient care and associated costs fall between those of ICUs and general wards. The patient-to-nurse ratio was 4:1 in high-dependency care units, 2:1 in ICUs, and 7:1 in general wards.

In this study, we excluded two specific groups of patients to minimize any potential bias based on prior research (16–18). First, the patients who died within two days of admission were excluded because they were unlikely to have survived, irrespective of the ward to which they were admitted, and this group was more likely to include patients for whom treatment was withdrawn. Second, patients with hospital stays longer than 60 days were excluded because our study focused on acute-phase patients and did not target those requiring prolonged hospitalization.

We categorized the children identified using mechanical ventilation into two groups. The PICU group consisted of children who were ventilated only in PICUs, and the GICU (non-PICU) group included children who were ventilated only in GICUs. Patients who were treated in both ICUs were also excluded.

In the present study, ICUs were defined as separate units providing critical care services, staffed by at least one in-house physician 24 h a day, and with a nurse-to-patient ratio of one nurse per two patients. According to the Japanese government insurance policy, patients in PICUs must be ≤14 years of age. The target conditions of PICUs are an impaired consciousness or coma, acute respiratory failure or an acute exacerbation of chronic respiratory failure, acute heart failure, acute drug poisoning, shock, severe metabolic disorders, extensive burns, severe trauma, post-major surgery, post-resuscitation, and other critical conditions.

Patients were classified into the following three groups based on mechanical ventilation: tracheostomy, non-invasive positive pressure ventilation, and intubation. Patients who were intubated at least once were included in the intubation group. All patients with tracheostomies were managed with positive-pressure ventilation. Patients who received only nasal high-flow therapy were excluded from the study.

### Patient backgrounds and outcomes

The patient characteristics were determined based on previous research (19) and included age, sex, body weight at admission, the Japan Coma Scale (JCS) (20) at admission, pediatric complex chronic condition classification system (version 2) (21), ambulance use, emergency admission, weekend admission, pre-admission facility, home medical care prior to admission, disease type, operation type, year of hospitalization, and classification of mechanical ventilation (intubation, tracheostomy, and non-invasive positive pressure ventilation). The following procedures were performed within two days of admission: arterial line, central venous line, renal replacement therapy, extracorporeal membrane oxygenation, nitric oxide inhalation, sedation, opioids, muscle relaxants, enteral nutrition, vasoactive drugs (dopamine, dobutamine, noradrenaline, adrenaline, vasopressin, and milrinone), blood transfusions (red blood cells, fresh frozen plasma, and platelets), albumin, defibrillation, and chest compression. The hospital characteristics included the type of hospital and hospital volume (22).

The primary outcome was all-cause in-hospital mortality. The secondary outcomes included the length of ICU and hospital stay and total hospitalization costs (with an exchange rate of 1 US dolla*r* = 150 Japanese yen).

### Statistical analyses

We herein describe the patient background, procedures, and hospital characteristics of the PICU and GICU groups. Continuous variables are presented as means with standard deviations (SDs), and categorical variables are summarized as frequencies and percentages.

We conducted overlap weighting (OW), a propensity score weighting method, to compare the outcomes between the PICUs and GICUs (23). OW is a recently developed propensity score weighting approach, and its details have been described elsewhere (24, 25). Briefly, unlike propensity score matching, which often targets the average treatment effect in the treated, OW targets the average treatment effect in the overlap population. By assigning smaller weights to individuals with extreme propensity scores and larger weights to those in the region of clinical equipoise, OW can achieve excellent covariate balance, which corresponds to the exact balance in the means of the included covariates when propensity scores are estimated using logistic regression. In contrast to propensity score matching, OW retains all individuals in the weighted analysis, thereby avoiding the exclusion of unmatched subjects and often improving the statistical efficiency. Because OW uses bounded weights and avoids arbitrary analytic choices, such as caliper selection in matching, it can also provide more stable treatment-effect estimates in observational studies. These features make the OW a useful and efficient alternative to propensity score matching when the estimand of interest is the treatment effect in the overlap population.

The propensity score for PICU admission was estimated using a multivariable logistic regression model incorporating all patient background and hospital characteristics, and the OW weights were derived from the estimated propensity scores. To assess covariate balance, we calculated the standardized mean differences between the groups for each covariate before and after weighting; an absolute standardized mean difference of <10% was considered indicative of a negligible imbalance. In the weighted population, we estimated the odds ratios for in-hospital mortality and mean differences in the ICU length of stay and total hospital length of stay. Corresponding 95% confidence intervals were calculated using generalized estimating equations, with hospital facilities treated as clusters and an identity link function.

#### Subgroup analyses

We categorized the patients into four groups (nonoperative, operative, intubated, and those who underwent tracheostomy) and compared the in-hospital mortality between the PICUs and GICUs in the same manner.

#### Sensitivity analyses

As sensitivity analyses, we repeated the primary analysis, including patients who died within 2 days of admission or who had a total hospital stay exceeding 60 days, who were excluded from the primary analysis. Additionally, we performed an analysis restricted to prefectures where both PICUs and GICUs were present, excluding 26 prefectures without a PICU.

All analyses were conducted using the Stata/MP 18.0 software program (Stata Corp LP, College Station, TX, USA). All reported *p*-values were two-sided, with *p* < 0.05 considered to be statistically significant.

## Results

A patient selection flowchart is presented in Figure 1. The characteristics of the children treated in PICUs and GICUs are summarized in Table 1. A total of 48,106 eligible patients were included in the analysis, of whom 17,085 (36%) received care in PICUs and 31,021 (64%) in GICUs. Among the GICUs, 55% were located in university hospitals. In both groups, children aged ≤6 years accounted for approximately 80% of admissions, and nearly 90% were managed with endotracheal intubation. Medical admissions accounted for 48% of PICU patients and 41% of GICU patients, whereas surgical admissions accounted for 52% and 59%, respectively (Table 1).

**Table 1:** Baseline characteristics before and after propensity score overlap weighting analyses.

Variables	Unmatched cohort			Overlap weighted cohort		
	GICU *n* = 31,021(%)	PICU *n* = 17,085(%)	SD (%)	GICU (%)	PICU (%)	SD (%)
Age, years						
<1y	13,136 (42)	7,285 (42)	0.0	(46)	(46)	0.0
1–6y	12,439 (40)	7,298 (43)	−5.5	(41)	(41)	0.0
7–9y	2,152 (7)	1,132 (7)	0.4	(5.9)	(5.9)	0.0
10–14y	3,294 (11)	1,370 (8)	8.9	(7.8)	(7.8)	0.0
Male	16,591 (53)	9,271 (54)	−1.9	(54)	(54)	0.0
Body weight, mean(SD)	12.2 (12.5)	11.2 (9.5)	1.8	10.9	10.9	0.0
Respiratory status						
Intubation	27,459 (89)	14,476 (85)	11.0	(89)	(89)	0.0
Tracheostomy	3,225 (10)	2,230 (13)	−8.3	(10)	(10)	0.0
Noninvasive positive pressure ventilation	337 (1)	379 (2)	−8.9	(1)	(1)	0.0
Pediatric complex chronic condition						
Neuromuscular	1,724 (5.0)	1,230 (7.0)	−7.6	(5.0)	(5.0)	0.0
Cardiovascular	11,202 (36)	6,037 (35)	1.7	(42)	(42)	0.0
Respiratory	784 (2.0)	796 (5.0)	−13.3	(3.0)	(3.0)	0.0
Renal	180 (1.0)	180 (1.0)	−5.2	(1.0)	(1.0)	0.0
Gastrointestinal	632 (2.0)	580 (3.0)	−8.9	(2.0)	(2.0)	0.0
Hema/immunology	332 (1.0)	156 (1.0)	1.6	(1.0)	(1.0)	0.0
Metabolic	1,426 (5.0)	932 (5.0)	−4.3	(4.0)	(4.0)	0.0
Congenital/genetic	2,415 (8.0)	1,461 (8.0)	−3.2	(8.0)	(8.0)	0.0
Malignancy	363 (1.0)	171 (1.0)	0.6	(1.0)	(1.0)	0.0
Neonatal	153 (0.0)	133 (1.0)	−4.3	(1.0)	(1.0)	0.0
Technology Dependent	515 (2.0)	624 (4.0)	−14.1	(2.0)	(2.0)	0.0
Weekend admission	4,117 (13)	1,986 (12)	4.5	(11)	(11)	0.0
Japan coma scale						
Alert	26,219 (85)	13,766 (81)	11.6	(88)	(88)	0.0
Drowsy	1,364 (4)	1,016 (6)	−8.0	(3.6)	(3.6)	0.0
Somnolent	806 (3)	634 (3)	−6.4	(2.2)	(2.2)	0.0
Comatose	2,632 (8)	1,669 (10)	−5.3	(6.1)	(6.1)	0.0
Transportation by ambulance	6,692 (22)	4,445 (26)	−10.7	(0.0)	(0.0)	0.0
Emergency admission	10,993 (35)	6,972 (41)	−13.4	(32)	(32)	0.0
Referral from another facility	19,931 (64)	11,025 (65)	−2.4	(68)	(68)	0.0
Academic	15,125 (55)	7,080 (44)	22.2	(45)	(45)	0.0
Hospital volume (pediatric annual admission)						
Lowest	3,340 (11)	2 (0)	49.8	(0.0)	(0.0)	0.0
Low	6,583 (23)	3 (0)	77.8	(0.0)	(0.0)	0.0
Middle	8,130 (26)	2,207 (13)	31.2	(25)	(25)	0.0
High	6,462 (21)	5,249 (31)	−22.8	(38)	(38)	0.0
Highest	6,506 (19)	9,624 (56)	−82.2	(38)	(38)	0.0
Admission path						
Home	27,891 (90)	14,497 (85)	15.5	(90)	(90)	0.0
Other hospital	2,991 (10)	2,483 (15)	−15.2	(10)	(10)	0.0
Nursing home	76 (0)	68 (0)	−2.8	(0.0)	(0.0)	0.0
Internal medicine and Surgery						
Internal medicine						
respiratory	3,907 (30.4)	2,520 (30.4)	0.0	(26.3)	(26.3)	0.0
neonate	3,676 (28.6)	2,417 (29.2)	−1.3	(39.4)	(39.4)	0.0
neurological	1,598 (12.4)	1,052 (12.7)	−0.8	(9.7)	(9.7)	0.0
cardiac	1,124 (8.8)	781 (9.4)	−2.4	(9.8)	(9.8)	0.0
endocrine	229 (1.8)	155 (1.9)	−0.7	(1.4)	(1.4)	0.0
infection	543 (4.2)	403 (4.9)	−3.1	(3.5)	(3.5)	0.0
cancer	565 (4.4)	239 (2.9)	8.1	(3.0)	(3.0)	0.0
gastric	135 (1.1)	105 (1.3)	−2.0	(1.0)	(1.0)	0.0
Others	1,064 (8.3)	607 (7.3)	3.6	(5.8)	(5.8)	0.0
Surgery						
cardiac	13,077 (71.9)	5,738 (65.2)	14.6	(77.6)	(77.6)	0.0
brain	1,532 (8.4)	606 (6.9)	5.8	(5.2)	(5.2)	0.0
orthopedic	459 (2.5)	221 (2.5)	0.1	(1.5)	(1.5)	0.0
pediatric	1,732 (9.5)	1,175 (13.3)	−12.0	(9.7)	(9.7)	0.0
others	1,380 (7.6)	1,066 (12.1)	−15.2	(6.0)	(6.0)	0.0
Procedure within two days after admission						
Arterial line	8,721 (26)	6,064 (36)	−23.6	(26)	(26)	0.0
Central venous line	6,017 (18)	4,603 (27)	−21.9	(19)	(19)	0.0
Hemodialysis	283 (1)	176 (1)	−1.7	(1.0)	(1.0)	0.0
Extracorporeal membrane oxygenation	135 (0)	99 (1)	−2.9	(0.0)	(0.0)	0.0
Nitric oxide	340 (1)	219 (1)	−4.2	(1.0)	(1.0)	0.0
Sedation	12,753 (39)	8,533 (50)	−23.4	(38)	(38)	0.0
Opioid	9,436 (28)	7,394 (44)	−32.8	(30)	(30)	0.0
Muscle relaxants	8,835 (26)	7,226 (43)	−34.7	(29)	(29)	0.0
Enteral nutrition	4,134 (13)	3,878 (22)	−25.4	(15)	(15)	0.0
Catecholamines	6,160 (18)	3,873 (23)	−12.5	(19)	(19)	0.0
Defibrillation	80 (0)	53 (0)	−0.8	(0.0)	(0.0)	0.0
Chest compression	363 (1)	145 (1)	3.0	(1.0)	(1.0)	0.0
Fresh frozen plasma	3,147 (9)	1,862 (11)	−6.6	(10)	(10)	0.0
Packed red blood Cells	4,524 (13)	2,738 (16)	−8.3	(14)	(14)	0.0
Platelets	1,611 (5)	821 (5)	0.3	(5.0)	(5.0)	0.0
Albumin	3,666 (11)	2,602 (15)	−13.7	(13)	(13)	0.0
Fiscal year						
2010	1,328 (4.3)	611 (3.6)	3.6	(4.9)	(4.9)	0.0
2011	1,838 (5.9)	1,051 (6.2)	−1.0	(7.1)	(7.1)	0.0
2012	2,571 (8.3)	1,232 (7.2)	4.0	(8.8)	(8.8)	0.0
2013	2,318 (7.5)	1,426 (8.3)	−3.2	(8.6)	(8.6)	0.0
2014	2,959 (9.5)	1,859 (10.9)	−4.4	(9.8)	(9.8)	0.0
2015	2,999 (9.7)	1,867 (10.9)	−4.1	(9.7)	(9.7)	0.0
2016	2,985 (9.6)	1,948 (11.4)	−5.8	(9.8)	(9.8)	0.0
2017	3,130 (10.1)	1,514 (8.9)	4.2	(9.4)	(9.4)	0.0
2018	3,070 (9.9)	1,622 (9.5)	1.4	(8.9)	(8.9)	0.0
2019	2,966 (9.6)	1,366 (8.0)	5.5	(8.1)	(8.1)	0.0
2020	2,379 (7.7)	1,362 (8.0)	−1.1	(7.3)	(7.3)	0.0
2021	2,478 (8.0)	1,227 (7.2)	3.0	(7.6)	(7.6)	0.0

GICU, general intensive-care unit; PICU, pediatric intensive-care unit; SD, standardized difference.

Positive values of the SD indicate higher values in the GICU group than in the PICU group.

The categories “Internal medicine” and “Surgery” in the table show the percentage distribution of cases within each group, with the total for each category summing to 100%. Classification of internal medicine-based on ICD-10: Respiratory disorder, J00-J99; Disorder of Neonatal origin, P00-P96, Q00-Q99; Neurological Disorder, G00-G99; Endocrine Disorders, E00-90; Cardiovascular Disorder, I00-I99; Infection, A00-B99; Cancer, C00-C97; Gastrointestinal disorder, K00-K93.

Among the non-operative patients, respiratory, neonatal, and neurological diseases constituted the majority of diagnoses in both PICUs and GICUs. Among the operative patients, cardiac and pediatric surgical cases predominated in both settings (Table 1). The use of arterial lines, central venous lines, sedatives, analgesics, neuromuscular blocking agents, and enteral nutrition was more common in PICUs than in GICUs (Table 1).

The baseline characteristics were well balanced between the two groups after applying OW. Table 2 summarizes the outcomes before and after OW. After OW, mechanically ventilated children treated in PICUs had significantly lower in-hospital mortality than those treated in GICUs (mean 2.73% vs. 3.42%; odds ratio, 0.79; 95% CI, 0.69–0.91; *p* = 0.001). The length of hospital stay was significantly longer in PICUs than in GICUs (mean 22.74 vs. 22.13 days; difference, +0.61 days; 95% CI, 0.3–0.9; *p* < 0.001). PICU patients also had longer ICU stays than GICU patients (mean 6.37 vs. 5.42 days; difference, +0.95 days; 95% CI, 0.82–1.09; *p* < 0.001). The total hospitalization costs were also higher in the PICU group than in the GICU group (mean $26,973 vs. $26,203; difference, +$770; 95% CI, $321–$1,219; *p* = 0.001).

**Table 2:** Outcomes before and after propensity score overlap weighting analyses.

Outcome	Before Overlap weighting		After Overlap weighting		Effect size	95% confidence interval	*P* value
	GICU	PICU	GICU	PICU			
	*n* = 31,021	*n* = 17,085					
In-hospital mortality	1,242 (4.00%)	532 (3.11%)	(3.42%)	(2.73%)	0.79^a^	0.69 to 0.91	=0.001
Length of hospital stay (days, Mean ± SD) ICU stay (days, Mean ± SD)	22.11 ± 12.81 5.47 ± 4.70	21.19 ± 13.57 6.31 ± 5.34	22.13 5.42	22.74 6.37	+0.61^b^ +0.95^b^	0.30 to 0.93 0.82 to 1.09	<0.001 <0.001
Total hospital costs (USD, Mean ± SD)^c^	24,820 ± 19,597	23,627 ± 17,845	26,203	26,973	+770^b^	321 to 1219	=0.001

GICU, general intensive-care unit; PICU, pediatric intensive-care unit; SD, Standard Deviation.

a Odds ratio.

b Difference.

c As of October 6, 2025, the exchange rate was 150 Japanese yen per US dollar. This information is based on data from Titan FX Research Hub website (https://research.titanfx.com/historical-rates/usdjpy/2025/10/06?utm_source=chatgpt.com), which was accessed on October 6, 2025.

Subgroup analyses were performed stratified by intubation status and surgical intervention; after OW, all covariates achieved a perfect balance across all subgroups (Supplementary Figures S1–S4; all absolute standardized differences = 0). As shown in Table 3, PICUs were associated with significantly lower in-hospital mortality among intubated patients (odds ratio, 0.79; 95% CI, 0.69–0.91; *p* = 0.001) and among patients who underwent surgery (odds ratio, 0.72; 95% CI, 0.57–0.91; *p* = 0.006).

**Table 3:** Subgroup analyses for in-hospital mortality of general ICU patients compared with pediatric ICU patients in the propensity score overlap weighted population.

Subgroup	Before Overlap weighting		After Overlap weighting		Odds ratio	95% confidence interval	*P* value
	GICU	PICU	GICU	PICU			
	*n* = 31,021	*n* = 17,085					
Non-operative patients	730/12,841 (5.68%)	359/8,279 (4.34%)	(4.75%)	(4.27%)	0.89	0.74 to 1.08	=0.247
Operative patients	512/18,180	173/8,806			0.72	0.57 to 0.91	=0.006
	(2.82%)	(1.96%)	(2.46%)	(1.79%)			
Intubated patients	1,233/27,459	525/14,476			0.79	0.69 to 0.91	=0.001
	(4.49%)	(3.63%)	(3.84%)	(3.06%)			
Tracheostomy patients	3/3,225	0/2,230			-	-	-
	(0.09%)	(0.00%)	(0.02%)	(0%)			

GICU, general intensive-care unit; PICU, pediatric intensive-care unit.

As shown in Table 4, both sensitivity analyses yielded results consistent with the primary analysis results. In the analysis including patients who died within 2 days or had a total hospital stay exceeding 60 days, in-hospital mortality was 4.64% in the GICU group and 3.87% in the PICU group (aOR 0.83, 95% CI 0.74–0.93, *P* = 0.001). In the analysis restricted to prefectures with both GICUs and PICUs, mortality was 3.38% in the GICU group and 2.60% in the PICU group (aOR 0.76, 95% CI 0.65–0.90, *P* = 0.001).

**Table 4:** Sensitivity analyses for in-hospital mortality.

Subjects	GICU (%)	PICU (%)	aOR^a^	95% CI	*P* value
Subjects including death ≤ 2 days and total hospital days > 60 days	4.64	3.87	0.83	0.74 to 0.93	＝0.001
Subjects excluding 26 prefectures without PICU	3.38	2.60	0.76	0.65 to 0.90	＝0.001

aOR, adjusted odds ratio; GICU, general intensive care unit; PICU, pediatric intensive care unit; CI, confidence interval.

a Adjusted odds ratios were estimated using overlap weighting to balance measured confounders between the GICU and PICU groups.

## Discussion

In this nationwide analysis of > 48,000 mechanically ventilated children, we observed substantial differences in patient characteristics, care practices, and outcomes between PICUs and GICUs. Although most patients in both settings were young children and were similarly managed with endotracheal intubation, PICUs treated a slightly higher proportion of medical cases and employed invasive monitoring, sedation, and nutritional support more frequently.

After OW, treatment in PICUs was associated with significantly lower in-hospital mortality than treatment in GICUs, despite longer ICU and hospital stays and slightly higher costs. These findings were consistent with the subgroup analyses of intubated and surgical patients. Overall, these findings suggest that the specialized expertise, infrastructure, and multidisciplinary care available in PICUs contribute to improved survival among critically ill children requiring mechanical ventilation (26).

To date, only a Finnish nationwide study (6) has demonstrated a clear mortality advantage of PICUs over GICUs. In contrast, Swedish data (5) showed low mortality in both settings without significant differences between them. Studies from Australia-New Zealand (7) and South Korea (26) also favored PICUs, although this was largely attributable to the declining PICU mortality over time.

Studies from Sweden, Australia-New Zealand, and Finland (5–7) have consistently reported longer ICU stays in PICUs, similar to our findings. Comparative data on total hospital costs are unavailable, and cost differences are likely influenced by national reimbursement systems; therefore, our findings may not be generalizable.

Based on our findings and previous reports (5–7, 26), the centralization of critically ill children in PICUs appears to be desirable. However, PICU capacity remains limited in Japan and many Asian countries (8–11), making rapid expansion difficult in the future. Accordingly, improving outcomes for mechanically ventilated children treated outside PICUs remains a challenge.

Japan is also facing rapid population aging and declining birth rates (27, 28). Currently, 26 of 47 prefectures lack a PICU, and many regions no longer have sufficient patient volumes to sustain a six-bed PICU. Population projections suggest that pediatric populations outside metropolitan areas will halve within 25 years (28). Although interregional transport may partially address this issue (29), limited transport resources make reliance on long-distance transfers unrealistic.

Our previous study (12) demonstrated better outcomes in pediatric and GICUs than in the general wards. Taken together, these findings suggest that strengthening pediatric critical care within existing GICUs may be a pragmatic strategy for improving outcomes. Potential measures include the deployment of PICU-trained physicians (30) and remote specialist consultation systems (31).

### Limitations

This study had several limitations. First, causality cannot be established because of its observational design. Second, the DPC database lacks detailed clinical information, including severity scores (PIM2, PIM3, and p-SOFA), ventilator settings, physiological variables, and treatment-limitation decisions (32, 33). Consequently, residual confounding may remain, despite propensity score adjustment. Nevertheless, the propensity score model incorporated multiple surrogate markers of illness severity, and overlap weighting improved the covariate balance between groups (34). Similar approaches have been used in previous administrative database studies (12, 35–37). Third, although the DPC database is nationwide, it does not include all hospitals in Japan. Fourth, we did not adjust the total hospitalization costs for inflation because the hospitalization costs in the DPC database are based on reimbursement points determined by the national fee schedule. However, we additionally adjusted for the fiscal year in the regression model to account for temporal changes in reimbursement and practice patterns. Fifth, only 36% of patients were treated in PICUs, reflecting the limited PICU capacity and incomplete centralization of pediatric critical care in Japan. The relatively small PICU sample reduced the statistical precision, resulting in wider confidence intervals. Moreover, because OW estimates treatment effects in the overlap population, our findings may not be generalizable to children treated in settings where PICU admission is not a realistic option.

## Conclusions

According to a nationwide inpatient database, more than half of the mechanically ventilated children in Japanese ICUs were treated in GICUs rather than in PICUs. Care in GICUs was associated with higher in-hospital mortality relative to that in PICUs. These findings support the centralization of pediatric critical care in PICUs. However, unmeasured confounding factors, including differences in illness severity and treatment-limitation decisions, could not be fully addressed. Therefore, further studies are required to confirm these results.

## Data Availability

The original contributions presented in the study are included in the article/Supplementary Material, further inquiries can be directed to the corresponding author.
