# Supplementary material for: Patient backgrounds and outcomes of mechanically ventilated children treated in pediatric intensive-care units versus general intensive-care units in Japan: a retrospective cohort study using a national inpatient database
Source: Front Pediatr. 2026 Jul 29;14:1881384. doi: 10.3389/fped.2026.1881384 (PMC13462237; doi:10.3389/fped.2026.1881384)
Supplement: Supplementary file 1 [file Supplementaryfile1.doc]

**Supplement data**

We thank the reviewer for this important point. As noted above, overlap weighting was applied within each subgroup, and covariate balance was confirmed for all subgroups. Absolute standardized differences were 0 for all covariates after weighting, as shown in Supplemental Figures 1–4.

s-Fig1. Covariate balance of internal medicine subgroup before and after overlap weighting

s-Fig2. Covariate balance of operative subgroup before and after overlap weighting

s-Fig3. Covariate balance of intubation subgroup before and after overlap weighting

s-Fig4. Covariate balance of tracheostomy subgroup before and after overlap weighting
